# Increased frequency of activated CD8^+^ T cell effectors in patients with psoriatic arthritis

**DOI:** 10.1038/s41598-019-47310-5

**Published:** 2019-07-26

**Authors:** Marco Diani, Fabio Casciano, Laura Marongiu, Matteo Longhi, Andrea Altomare, Paolo D. Pigatto, Paola Secchiero, Roberto Gambari, Giuseppe Banfi, Angelo A. Manfredi, Gianfranco Altomare, Francesca Granucci, Eva Reali

**Affiliations:** 1 IRCCS Istituto Ortopedico Galeazzi, Milan, Italy; 2 Department of Morphology, Surgery and Experimental Medicine and LTTA Centre, University of Ferrara, Ferrara, Italy; 3 Department of Biotechnology and Biosciences, University of Milano-Bicocca, Milan, Italy; 4 University of Milan, Milan, Italy; 5 Department of Life Sciences and Biotechnology, University of Ferrara, Ferrara, Italy; 6 Università Vita-Salute San Raffaele, Milan, Italy; 7 IRCCS Ospedale San Raffaele, Milan, Italy

**Keywords:** Autoimmunity, Skin diseases, Rheumatic diseases

## Abstract

The aim of this study is to identify subsets of T cells differentially represented in the circulation of patients with psoriatic arthritis and to evaluate the possibility that they can recirculate between peripheral blood and the inflamed joints. We analyzed the phenotype and cytokine expression in circulating CD8^+^ and CD4^+^ T cells in 69 subjects: 28 with cutaneous psoriasis, 15 patients with psoriatic arthritis, and 26 healthy subjects. In the circulation, the percentage of each subset was compared among the groups and correlation was calculated with the serum concentration of C-reactive protein. To investigate the migration of T cells towards the inflamed joints, we performed a transwell migration assay towards patient serum and synovial fluid. In selected patients we analyzed in parallel T cells from peripheral blood and from synovial fluid. In the circulation, we found increased percentage of CD8^+^ CCR6^+^ T cell effectors expressing CD69 and of IL-17-producing T cells in patients with psoriatic arthritis. CD8^+^ effector/effector memory T cells showed increased migration towards synovial fluid. Finally, in synovial fluid we found accumulation of CXCR3^+^ CD8^+^ T cells and CD69^+^ cells. CD4^+^ T cells in the two compartments shared many similarities with CD8^+^ T cells. The results indicate a role for memory T cell effectors in systemic and joint manifestations of psoriatic arthritis.

## Introduction

The most common manifestation of *psoriatic disease* is *psoriasis vulgaris*, a chronically relapsing skin inflammation affecting about 2% of the population worldwide. Psoriatic disease is associated with systemic inflammation and with comorbidities such as metabolic syndrome and cardioavascular disease. In addition 20–30% of patients with psoriasis also develop psoriatic arthritis (PsA) that in the majority of cases follows the cutaneous disease by a mean of 10 years^1–4^.

In the last decades, much progress has been made in the understanding of the mechanisms underlying the development of cutaneous psoriasis, highlighting a non-redundant interplay between epidermal keratinocytes, dendritic cells and T cells in the psoriatic plaque^5–7^. It is now clear that CD4^+^ T cells with a Th17 phenotype and other IL-17-producing lymphocyte subsets enhance the response of keratinocytes by creating a positive feedback loop around the IL-23/IL-17 axis^5,6,8–10^.

In the last years CD8^+^ T cells have attracted much attention after the identification, in patients with psoriasis, of autoreactive T cells specific for melanocyte-derived antigens presented by HLA-C*0602 class I molecules and other self-antigens recognized by T cells^11–13^.

Unlike psoriasis, the immunopathogenic mechanisms leading to the development of PsA are poorly characterized and the link between the T cell responses arising in the skin and the development of joint manifestations is still largely unknown.

Genetic association studies have shown that PsA patients, in addition to the association with HLA-C*0602 allele that is shared with cutaneous psoriasis, have higher frequency of haplotype containing HLA-B*8, HLA-B*27, HLA-B*38 and HLA-B*39 pointing towards a role for CD8^+^ T cell and HLA class I-dependent mechanisms in the development of joint manifestations^14,15^.

In patients with cutaneous psoriasis, we previously found that the frequency of CD8^+^ T cell effectors expressing CCR4 and CD103 in the circulation strongly correlated with both systemic inflammation and the severity of the disease. This has led to the hypothesis that CD8^+^ T cells recirculating from the skin may link the cutaneous manifestations of psoriasis with systemic inflammation^16–18^.

This study has the aim to identify in the circulation of patients with PsA, specific subsets of memory T cells with differential expression of chemokine receptors, cytokines and activation marker CD69. In addition it has the aim to find the correlation between each subset and systemic inflammation, to evaluate the migration of specific subsets of T cells towards PsA synovial fluid and to compare the phenotype of CD8^+^ and CD4^+^ T cells in peripheral blood and synovial fluid.

## Results

### Increased frequency of CD69^+^ CD8^+^ T cells in the circulation of patients with PsA

We have characterized the expression of the activation marker/tissue retention molecule CD69 and chemokine receptors in the circulating compartment of CD8^+^ and CD4^+^ T cells from patients with PsA, patients with cutaneous psoriasis without articular manifestations (PsO) and in healthy control subjects (Table 1 and Supplementary Table 1). The expression of the surface markers was analyzed in the total memory population (CD45RA^−^), in central memory T cells (CD45RA^−^CCR7^+^, T_CM_), in effector/effector memory T cells (CD45RA^−^CCR7^−^, T_EM_) and in effector memory CD45RA^+^ T cells (CD45RA^+^CCR7^−^, T_EMRA_).

**Table 1:** Demographic Characteristics of Patients and Controls.

Cohort	N	M	F	Age	PASI	CRP (mg/dL)	N of patients with Comorbidities	Comorbidities
PsO	26	18	8	45 ± 12.9	12 ± 4.7	0.34 ± 0.3	11	AA(1), HC(5), FP (1), AH (2), DC (1), RA (1), HT (1), CH (1), D (2)
PsA	15	12	3	51 ± 7.7	6.5 ± 5.9	0.68 ± 0.55	10	HC (5), HU (1), AH (3), DD (1), HS (1), PP (1), CH (1), HT (1), RU (1), FM (1), DS (1), IHD (1)
Controls	26	11	15	42 ± 11.3	0	ND	0	0

Age, PASI and CRP presented as mean ± SD. AH, arterial hypertension; CH, cholelithiasis; D, diabetes; DC, dilated cardiomyopathy; DD, depressive disorder; FM, fibromialgya; FP, familial poliposis; HC, hypercholesterolemia; HS, hepatic steatosis; HT, hypothyroidism; HU, hyperuricemia; IHD, ischemic heart disease; PP, previous pericarditis; RA, rhizarthrosis; RU, recurrent uveitis; NA, not applicable; ND, not determined.

**Figure 1:**
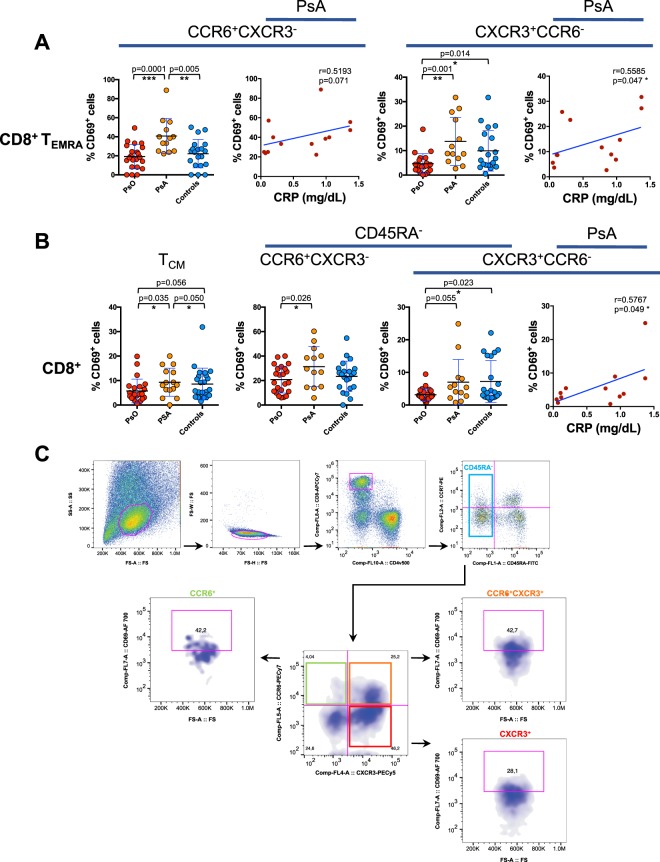
Increased frequency of CD69^+^ CD8^+^ T cells in the circulation of patients with PsA. (**A**) CD8^+^ T cells gated as CD45RA^+^CCR7^−^ T_EMRA_ were analyzed for the expression of CD69 in subsets of CD8 T cells expressing chemokine receptors CCR6 or CXCR3 in patients with psoriatic arthritis (PsA), patients without articular manifestations (PsO), and in control group of healthy subjects. Statistical analysis of the differences was performed by Mann-Whitney test. p values < 0.05 were considered significant: *p < 0.05, **p < 0.01, ***p < 0.001. (**B**) CD8^+^ T cells were analyzed for the expression of CD69 in CD45RA^−^CCR7^+^ T_CM_, and in CD45RA^−^ (total memory) according with the expression of chemokine receptors CCR6 or CXCR3. Correlations are expressed as Spearman r values, and significance level, calculated by Student’s t-test, are indicated in the figure. p values < 0.05 were considered significant: *p < 0.05. (**C**) Gating strategy used in multiparametric flow cytometry analysis of CD69 expressing cells in CD45RA^−^ CCR6/CXCR3 subsets. SS-A and FS-A axes are reported as linear values and fluorescences scales are reported as log.

In patients with PsA, we found a marked increase in the frequency of circulating CD8^+^ CCR6^+^ T_EMRA_ expressing CD69 compared to patients with cutaneous psoriasis and to healthy control subjects. Significant differences in CD69 expression compared to patients with cutaneous psoriasis were also observed in CXCR3^+^CCR6^−^ CD8^+^ T_EMRA_ cells (Fig. 1A). Importantly, in patients with PsA, there was a significant correlation betweeen systemic inflammation, measured as serum concentration of C-reactive protein and the percentage of CD69 expressing cells within CXCR3^+^ CD8^+^ T_EMRA_. With a similar trend, also CD69^+^ CCR6^+^ CD8^+^ T_EMRA_ positively correlated with serum concentration of C-reactive protein as well as with serum concentration IL-6 (Fig. 1A and Supplementary Fig. 1).

CD8^+^ T_CM_ from PsA patients cells also display significantly higher percentage of CD69^+^ cells compared to CD8^+^ T_CM_ from patients with psoriasis without articular involvement. Among the total CD8^+^CD45RA^−^ cells, the percentage of CD69^+^ cells was significantly higher in the CCR6^+^CXCR3^−^ subsets (Fig. 1B).

This data enlightens as a distinctive feature of PsA patients, an increased circulating level of CD8^+^ T cell effectors expressing the activated phenotype, which is associated with systemic inflammation.

### Expansion of IL-17^+^ and contraction of IFNγ^+^ cells in circulating memory T cells from patients with PsA

**Figure 2:**
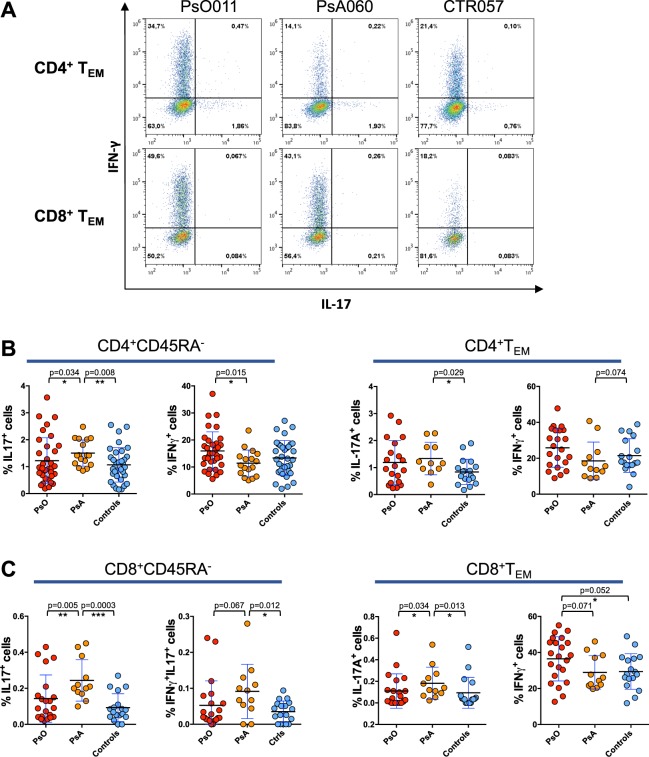
Expansion of IL-17^+^ and contraction of IFNγ^+^ cells in circulating memory T cells from patients with PsA. PBMCs isolated from PsA and PsO patients or from control subjects were stimulated with αCD3αCD28-coated beads. Cells were then evaluated for expression of IFNγ/IL-17 in CD45RA^−^ gated cells or CD45RA^−^CCR7^−^ T_EM_, CD4^+^ (**B**) and CD8^+^ T cells (**C**). Difference in the percentage of cytokine producing cells in the three groups was analyzed by Mann-Whitney test. p values < 0.05 were considered significant: *p < 0.05, **p < 0.01, ***p < 0.001. One representative dot plot experiment is shown in panel (**A**), the axis scales are reported as log.

We analyzed the expression of the cytokines IFNγ and IL-17 in circulating CD8^+^ and CD4^+^ memory T cells from patients with PsA, PsO and from control subjects upon stimulation with αCD3/CD28-coated beads. We found a significantly higher percentage of IL-17-producing CD8^+^CD45RA^−^ T cells in patients with PsA as compared to patients with PsO and control subjects (Fig. 2C). Similarly, IL-17^+^ cells were expanded in CD8^+^ T_EM_ and in CD4^+^ CD45RA^−^ cells from patients with PsA (Fig. 2B,C). IFNγ^+^IL-17^+^double producing CD8^+^ T cells were also significantly more frequent in PsA patients than in control subjects (Fig. 2C). By contrast we found that the frequency of IFNγ-producing CD4^+^CD45RA^−^ T cells in patients with PsA was lower than in patients with cutaneous psoriasis. Similar results were observed in both CD8^+^ and CD4^+^ T_EM_ subsets.

### Contraction of CXCR3^+^ subsets in circulating memory T cells from PsA patients

**Figure 3:**
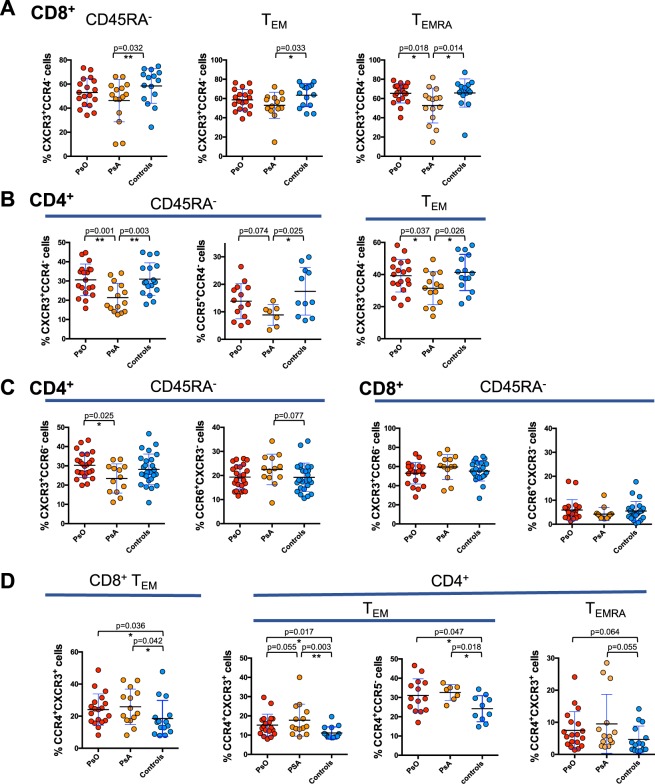
Contraction of CXCR3^+^ subsets in circulating memory T cells from PsA patients. PBMCs isolated from patients with PsA, PsO patients and control subjects were stained for CD4 and CD8 lineage markers, memory T cell phenotype markers (CD45RA and CCR7) and for chemokine receptors CCR4, CXCR3, CCR6 or CCR5. Percentages of T cells expressing CCR4, CXCR3, CCR6, CCR5 chemokine receptors in the total memory CD45RA^−^, T_EMRA_ and T_EM_ subsets of CD4^+^ and CD8^+^ T cells are shown in the figure. Statistical analysis of the differences was performed by Mann-Whitney test. p values < 0.05 were considered significant: *p < 0.05, **p < 0.01.

By analyzing the chemokine receptor profile in the different memory T cell subsets, we found a decrease in the circulating percentage of cells expressing the Th1/Tc1-associated trafficking receptor CXCR3 (CXCR3^+^CCR4^−^) in patients with PsA, both in the CD8^+^ and CD4^+^ compartment (Fig. 3A,B). The contraction of the CXCR3^+^ subpopulation was observed mainly in the CD8^+^ T_EMRA_ and in the CD4^+^ T_EM_ compartments. In patients with PsA, compared to control subjects we also found a decreased percentage of CD4^+^CD45RA^−^ T cells expressing CCR5, a marker of terminally differentiated Th1/Tc1 cells^19^.

The data indicates a decrease of cells with Th1/Tc1 phenotype within the CD8^+^ and CD4^+^ circulating memory T cell subsets of patients with PsA.

In PsA patients we also observed a moderate increase of cells expressing the Th17-Tc17 associated trafficking receptor CCR6 within the CD4^+^ CD45RA^−^ subset in line with the results of cytokine expression whereas no differences were observed in the CD8^+^ compartment (Fig. 3B). The percentage of CCR6^+^CXCR3^−^ cells within the CD4^+^ T_EM_ subset in the total cohort of psoriasis and PsA patients significantly correlated with serum concentration of CRP (Supplementary Fig. 2).

CCR4^+^ memory T cells have been previously suggested as a skin-to-blood recirculating subpopulation in cutaneous psoriasis^17^. Here we found that the frequency of CCR4^+^CXCR3^+^ in CD8 T_EM_ cells was increased in patients with PsA as compared to healthy subjects (Fig. 3D). However, their percentage was similar in patients with cutaneous psoriasis without articular manifestations. It is important to note that the percentage CCR4^+^CXCR3^−^ in CD8^+^ T_EM_ cells in the circulation of PsA patients was positively correlated with the serum concentration of CRP (Spearman r = 0.61, p = 0.02). Significant correlation with CRP was also shown by analyzing the entire cohort of patients with psoriatic disease (PsO and PsA) (Supplementary Fig. 3) and a trend towards a positive correlation was also found with serum concentration of IL-6 (r = 0.3, p = 0.06 not shown). This strengthens the hypothesis that CCR4 expressing CD8^+^ T cell effectors can contribute to the increase of systemic inflammation.

### Migration of CD8^+^ effector/effector memory T cells towards synovial fluid

To analyze the type of cells that migrate towards synovial fluid from PsA patients, we performed a transwell migration assay. We seeded PBMCs in the upper chamber and after 3 hour incubation at 37 °C, the CD8^+^ and CD4^+^ T cells in the lower chamber were analyzed to determine the expression of the chemokine receptors CXCR3 and CCR6.

**Figure 4:**
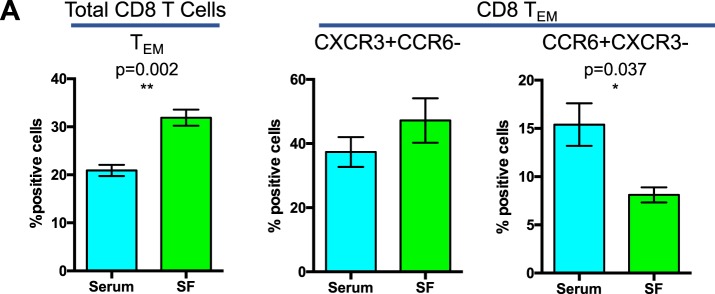
Migration of CD8^+^ effector/effector memory T cells towards synovial fluid. PBMCs isolated from control subjects were used for a transwell migration assay towards 10% serum or synovial fluid from 3 different PsA patients. Migrated cells were collected after 3 hours of incubation and analyzed for their CD4 and CD8 T_CM_, T_EM_ and T_EMRA_ phenotype, as well as for their expression of chemokine receptors CCR6 and CXCR3. Data represented the mean of positive cells ± SEM of 4 experiments performed with PBMCs from 2 different subjects, each tested for migration towards 2 different PsA serum-synovial fluid pairs. Statistical analysis of the differences was performed by Student’s t-test for paired samples. p values < 0.05 were considered significant: *p < 0.05.

Flow cytometric analysis indicates significantly increased migration of CD8^+^ T_EM_ cells towards synovial fluid from PsA patients as compared to their serum counterpart (Fig. 4).

In addition, within CD8^+^ T_EM_ cells migrated towards synovial fluid, we found a decrease in the percentage of CCR6^+^ cells and a trend towards an increase of the percentage of CXCR3^+^ cells.

### Accumulation of CXCR3 expressing T cells in the synovial fluid of patients with PsA

**Figure 5:**
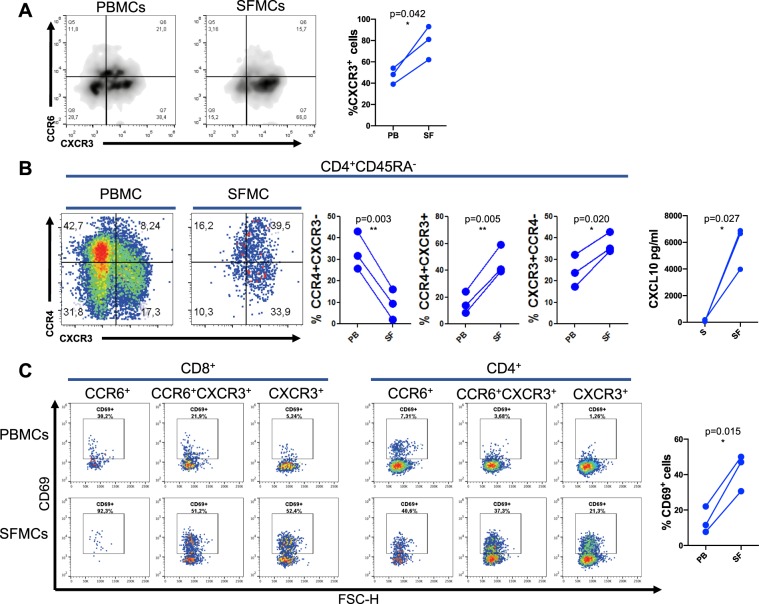
Accumulation of CXCR3^+^ T cells in the synovial fluid of patients with PsA. Mononuclear cells were isolated from peripheral blood and synovial fluids of patients with PsA and evaluated by flow cytometry. (**A**) PBMCs (PB) and SFMCs (SF) from three PsA patients were analyzed for the expression of chemokine receptor CXCR3 on total memory CD45RA^−^ CD8^+^ cells. Statistical analysis of the differences was performed by Student’s t-test for paired samples. p values < 0.05 were considered significant: *p < 0.05. Density plot from one representative patient with PsA analyzed for the expression of chemokine receptors CCR6 and CXCR3 in CD8^+^ CD45RA^−^ T cells is also shown in the panel. (**B**) PBMCs and SFMCs from three PsA patients were analyzed for the expression of chemokine receptor CCR4, CXCR3 in the CD4^+^CD45RA^−^ cell subsets, left side of the panel. Statistical analysis of the differences was performed by Student’s t-test for paired samples. p values < 0.05 were considered significant: *p < 0.05, **p < 0.01. One representative dot plot experiment is also shown in the panel, the axis scales are reported as log. CXCL10 concentration in serum (S) and paired synovial fluid (SF) samples from PsA patients is shown on the right side of panel. (**C**) PBMCs and SFMCs from three PsA patients were analyzed for the expression of CD69 activation marker on total memory CD45RA^−^ CD8^+^ from paired PBMCs and SFMCs. Statistical analysis of the differences was performed by Student’s t-test for paired samples. p values < 0.05 were considered significant: *p < 0.05. One representative dot plot analysis of CD69 expression on CD45RA^−^ CD8^+^ and CD4^+^ gated T cells from paired PBMCs and SFMCs is also shown in the panel, the axis scales are reported as log.

Finally, we analyzed the phenotypic differences between T cells in the blood and in the synovial fluid of patients with active PsA. Data in Fig. 5A show that in CD8^+^CD45RA^−^ compartment CXCR3^+^ T cells were significantly expanded in the inflamed joints as compared to peripheral blood. Accumulation of CXCR3^+^ cells in synovial fluid was observed also in the CD4^+^ T cell compartment (Fig. 5B). To support the hypothesis of a specific recruitment of CXCR3^+^ cells in the inflamed joint, we quantified the chemokine CXCL10, ligand of CXCR3, in serum and synovial fluid of PsA patients (Fig. 5B and Supplementary Fig. 4).

The results showed a marked enhancement of CXCL10 in synovial fluid as compared to their paired serum samples.

It is important to note that in synovial fluid there was a marked enhancement of CD69 expressing cells in CD8^+^CD45RA^−^ T cells as compared to peripheral blood regardless of the expression of chemokine receptors (Fig. 5C). The frequency of CD69^+^ cells was also increased in synovial fluid CD4^+^ T cells.

These results together indicate an accumulation of CXCR3^+^ T cells and cells with an activated phenotype in the inflamed joints of patients with psoriatic arthritis which is paralleled by an increase of the sinovial fluid concentration of the CXCR3-ligand chemokine CXCL10.

## Discussion

The phenotypic charaterization of circulating T cells in patients with psoriatic arthritis enlightened, as a distinctive feature, a selective enhancement of CD8^+^ T_EMRA_ expressing CD69. This increase was observed mainly in CCR6^+^ CD8^+^ T_EMRA_ and was associated with increased systemic inflammation measured as concentration of serum C reactive protein. In a previous study, in psoriasis patients, we found that circulating CCR4^+^ CD8^+^ T_EMRA_ expressing CD103 correlated with both systemic inflammation and the severity of cutaneous psoriasis^17^. In the present work, we show that the frequency of CCR4^+^ cells in the CD8^+^ T_EM_ subset positively correlated with serum concentration of CRP both by analyzing the entire cohort (PsO and PsA) and by analyzing separately the group of patients with PsA.

Together the data strengthen the hypothesis of a role for CD8^+^ T cells in an advanced stage of maturation as a putative link with systemic inflammation.

To investigate the possible role of CD8^+^ T cells in joint manifestations we performed an *in vitro* transwell migration assay which evidenced a preferential migration of CD8^+^ T_EM_ cells towards PsA synovial fluid. Moreover, parallel analysis of the T cell phenotype in patients peripheral blood and synovial fluid, revealed a markedly higher frequency of CD8^+^ T cells expressing the activation marker CD69 in synovial fluid as compared to peripheral blood. This is in line with the findings of a previous study by FitzGerald and coworkers showing that in patients with psoriatic arthritis, CD8^+^ T cells with a memory and activated phenotype are present at higher level in synovial fluid of PsA patients as compared to patients with rheumatoid arthritis^20^.

The results of our study evidence that PsA patients have, as a distinctive feature, an increased circulating frequency of CD8^+^ T cell effectors expressing an activated phenotype, which is associated with systemic inflammation, and is further enhanced in synovial fluid.

Analysis of the chemokine receptor profile at circulating level revealed that, in patients with PsA, there was a decrease in the percentage of CD8^+^ and CD4^+^ T cells expressing the Th1/Tc1 associated trafficking receptor CXCR3^+^ as compared to patients with cutaneous psoriasis and healthy subjects. PsA patients also showed a decrease in the circulating percentage of Th1/Tc1 IFNγ-producing CD8^+^ and CD4^+^ T cells as compared to patients with PsO. In a previous study performed in patients with PsO we found that CXCR3^+^ and CCR5^+^ CD4^+^ Th1 cells in the circulation inversely correlated with Psoriasis Area and Severity Index and that the expression of CXCR3 and CCR5 mRNA was increased in psoriatic skin as compared to normal skin leading to the hypothesis of a specific recruitment of CXCR3^+^ cells to the inflamed psoriatic tissues^16,17^. The concept of a recruitment of CXCR3^+^ T cell to the inflamed joints is supported by the analysis of the T cell phenotype in peripheral blood and synovial fluid mononuclear cells showing an accumulation of CXCR3^+^ T cells in synovial fluid. Importantly, we found a strong increase in the concentration of the CXCR3 ligand, CXCL10, in synovial fluid. This finding is in line with the marked up-regulation of CXCL10 mRNA that we observed in psoriatic skin and that other groups have reported in synovial fluid^16,17,21–25^. Therefore the CXCL10/CXCR3^+^ axis could represent a key downstream mechanism of inflammation in psoriatic skin and joints^26,27^.

T cell migration assay also showed that CD4^+^ and CD8^+^ T_EM_ migrated towards synovial fluid contained a lower percentage of CCR6^+^ cells and an increased percentage of CXCR3^+^ cells.

In this study we did not observe in synovial fluid from PsA patients the increase of cells with Th17/Tc17 phenotype that has been reported by Manon *et al*.^28^. However, it is increasingly evident that Th17 cells and the pathogenic IL-17/IFNγ-double expressing subset show high plasticity and may lose IL-17 expression to become Th1-like cells producing IFNγ^9,29^.

At circulating level, cells with IL-17-producing Th17/Tc17 phenotype as well as IL-17^+^IFNγ^+^ Th1* cells were increased in patients with PsA. Increased circulating IL-17-producing CD4^+^ T cells in PsA patients has been reported by Jandus *et al*., however a correlation with the systemic inflammation was not clearly established^30^.

In our cohort, analyzed either as patients with cutaneous psoriasis or as a larger group of patients with psoriatic disease, CD4^+^ T_EM_ cells expressing Th17-associated trafficking receptor CCR6 positively correlated with systemic inflammation (Supplementary Fig. 2)^16^.

CD69^+^ CCR6^+^ CD8^+^ T_EMRA_ and with CCR4^+^ CD8^+^ T_EM_ also showed correlation with the serum level of IL-6, one of the main regulator of CRP release by the liver. IL-6 is known to be augmented by IL-17 and IL-17 itself can directly induce CRP expression in hepatocytes^31–33^. Moreover, CD8^+^ T cell effectors, are equipped with powerful cytotoxic molecules such as granzyme and perforins and can produce IFNγ and TNF, other potential inducers of CRP^32^. Although it is not really possible to define in patients the mechanistic link between cytokine-producing T cell effectors and systemic inflammation, it is however conceivable that these factors together may link T cells effectors with smoldering tissue damage.

The results of this study underline the role of activated CD8^+^ T cell effectors in psoriatic arthritis and systemic inflammatory manifestations and indicate migration of CXCR3^+^ T cell from the blood to inflamed joint as a downstream event in the psoritic inflammatory cascade.

Understanding the link between T cell responses in skin and blood and those in inflamed joint could lead to the design of specific strategies for the successful prevention of PsA development.

## Materials and Methods

### Patients

Patients with psoriatic disease were recruited in the Department of Dermatology, Istituto di Ricovero e Cura a Carattere Scientifico Istituto Ortopedico Galeazzi (Milan, Italy). 69 subjects were studied, including 43 patients with psoriatic disease (28 patients with established cutaneous psoriasis without clinical signs of PsA and 15 patients with PsA) and 26 healthy subjects that served as controls. In three patients synovial fluid was collected in parallel with peripheral blood. Other systemic autoimmune diseases such as type 1 diabetes, neoplastic diseases, chronic or acute infections were used as exclusion criteria. Healthy controls had negative family and personal anamnesis for psoriasis. Subjects undergoing treatment with cyclosporin A, methotrexate, systemic corticosteroids or any other immunosuppressant or biotechnological agent within at least 3 weeks prior to the collection of blood samples were excluded from the study. Some demographic and clinical characteristics are summarized in Table 1 and Supplementary Table 1.

### Study design

We analyzed by flow cytometry the T cells subpopulations in patients with PsA, patients with psoriasis without articular manifestations and in healthy controls. We characterized the total memory population (CD45RA^−^), the “central memory” T cells (CD45RA^−^CCR7^+^, T_CM_), the “effector/effector memory” T cells (CD45RA^−^CCR7^−^, T_EM_) and the “effector memory CD45RA^+^” T cells (CD45RA^+^CCR7^−^, T_EMRA_) in both the CD4^+^ and CD8^+^ compartment. Within these subsets we analyzed the expression of the chemokine receptors CXCR3, CCR6, CCR4 and CCR5 and of the activation marker CD69. The expression of the cytokines, IFNγ and IL-17 after activation of the CD3/CD28 complex was also evaluated. We correlated the percentage of each population with the extent of systemic inflammation measured as serum level of C reactive protein.

We then set up a migration assay to identify the subpopulations of circulating T cells that migrate towards patient synovial fluid and finally we compared the phenotype of T cells in peripheral blood and synovial fluid of three patients.

### Multiparameter flow cytometry analysis and Intracellular Cytokine Staining

Blood samples were harvested from each patient and control into BD Vacutainer tube (BD Biosciences) containing EDTA. Peripheral blood mononuclear cells (PBMCs) were isolated from whole blood from patients or healthy controls by Ficoll gradient centrifugation (Lympholyte®, Cederlane® Hornby, Ontario, Canada) as previously described^34^. Cells were subsequently frozen and stored in liquid nitrogen for subsequent analysis. Analysis of samples from patients with PsA, patients with PsO and from healthy donors were carried out in parallel (usually one for each group). PBMCs were thawed, incubated overnight in complete medium and either stained for surface marker detection or stimulated for intracellular cytokine expression. For the detection of the intracellular cytokine expression, PBMCs were incubated with or without αCD3αCD28-coated beads 1:1 cells:beads (Dynabeads® Human T-Activator CD3/CD28, Invitrogen™). Brefeldin-A (GolgiPlug, BD biosciences) was added after 2 hours and cells were incubated overnight for 14 hours. Synovial fluid mononuclear cells (SFMCs) were isolated following the same protocol and analyzed after thawing, together with paired PBMCs samples.

Surface and intracellar staining of both unstimulated and αCD3αCD28-stimulated SFMCs and PBMCs were performed according to standard protocols upon FcR-blocking with human serum. We used combinations of fluorochrome-conjugated antibodies against CD4 (RPA-T4), CD8 (SK1), CCR7 (3D12), CD45RA (HI100), CCR6 (11A9), CXCR3 (1C6), CCR5 (2D7/CCR5), CCR4 (1G1), CD69 (FN50), CLA (HECA-452), IFNγ (B27) and IL-17 (SCPL1362) (BD Biosciences, Franklin Lakes, NJ, USA). The panels with antibody and fluorochromes used for each staining are listed in Supplementary material 1. Briefly, for surface staining, cells were incubated at RT for 20′ with antibodies against the chemokine receptors and CLA, then antibodies against the lineage markers CD3, CD4, CD8, CD45RA and CD69 were added and incubated at 4 °C for 20′. For Intracellular Cytokine Staining (ICS) cells stained for surface markers were incubated with anti-cytokine antibodies at 4 °C for 30 minutes in Cytofix/Cytoperm™ solution (BD Biosciences, Franklin Lakes, NJ, USA) according to manufacturer’s indications and then analyzed for intracellular detection of IFNγ and IL-17. Appropriate isotype controls were used for each staining. In order to assess fluorescence compensation, cells stained with the single antibodies were used. In each acquisition at least 100,000 of lympho gate events were recorded. All data were collected on a Gallios Flow Cytometer using Kaluza Software (Beckman Coulter, Brea, CA). Data were analyzed with the FlowJo software (Tree Star, Ashland, OR).

### Transwell migration assay

Human PBMCs were resuspended in RPMI 1640 medium containing 0.1% Bovine Serum Albumin (BSA) and seeded in the upper chamber of a 3-µm pore size Transwell plate (Corning Incorporated, Corning, NY, via Fisher Scientific, Pittsburgh, Pa), 3 × 10^5^ cells/well^35^. The lower chambers were filled with RPMI 1640 medium supplemented with 0.1% BSA added with 10% serum or synovial fluid from PsA patients. Controls were performed using 10% control serum from healthy subjects in the lower chamber. After 3 hours at 37 °C, the phenotype of the cells that had migrated into the lower chamber was evaluated by flow cytometry. Cells and compensation controls were stained as described above and samples were acquired using a FACS ARIA II and FACS DIVA sofware (BD Biosciences, Franklin Lakes, NJ, USA). Data were analyzed with the FlowJo software (Tree Star, Ashland, OR).

### Serum collection and quantification of C reactive protein, CXCL10 and IL-6

Serum was collected from blood samples from patients and controls, and stored at −80 °C. Serum was then tested for C reactive protein quantification by CRP assay performed by Architect c8000 clinical chemistry analyzer (Abbott, Italy).

For the detection of soluble CXCL10 and IL-6 in serum and synovial fluid samples IL-6 and CXCL10 duo-set® ELISA kit (R&D Systems, Minneapolis, MN) were used according to the manufacture’s instructions.

### Statistical analysis

Statistics were calculated with Graphpad prism 6 software.

The Shapiro-Wilk test was used to evaluate the Gaussian distribution of overall data. Statistical comparisons between the different groups of patients and healthy controls were then calculated with non-parametric analyses (Mann–Whitney non-parametric U-test) when no Gaussian distribution was found and exact p values were obtained, otherwise Student’s t-test was used. All data in scatter plot are given as mean value and standard deviation.

Correlation among variables was evaluated using the Spearman’s rank correlation coefficient or Pearson’s correlation coefficient according to the Gaussian distribution of the data.

### Ethics statement

The study was approved by the local Ethical Committee (Comitato Etico dell’Ospedale San Raffaele, Milan, Italy) (30IOG 17/07/2014), and written informed consent was obtained from all patients and healthy subjects before they entered the study, which was performed in accordance with the Declaration of Helsinki. The study was registered on ClinicalTrials.gov, Identifier: NCT03374527.

## Supplementary information

Supplementary Info

## Data Availability

All authors confirmed the availability of data and materials upon request.
