# Supplementary material for: Increased frequency of activated CD8+ T cell effectors in patients with psoriatic arthritis
Source: Sci Rep. 2019 Jul 26;9:10870. doi: 10.1038/s41598-019-47310-5 (PMC6659700; doi:10.1038/s41598-019-47310-5)
Supplement: Supplementary file 1 — Supplementary Info [file 41598_2019_47310_MOESM1_ESM.pdf]

Increased frequency of activated $CD8^+$ T cell effectors in patients with psoriatic arthritis.

Marco Diani[1], Fabio Casciano[2], Laura Marongiu[3], Matteo Longhi[4], Andrea Altomare [1], Paolo D. Pigatto[1], Paola Secchiero[2], Roberto Gambari[5], Giuseppe Banfi[1, 4, 7], Angelo A. Manfredi[6], Gianfranco Altomare[1], Francesca Granucci[3] and Eva Reali[5, 7 *]

[1] Department of Dermatology and Venereology, I.R.C.C.S Istituto Ortopedico Galeazzi, University of Milan, Milan, Italy.
[2] Department of Morphology, Surgery and Experimental Medicine and LTTA Centre, University of Ferrara, Ferrara, Italy.
[3] Department of Biotechnology and Biosciences, University of Milano-Bicocca, Milan, Italy.
[4] Department of Rheumatology, I.R.C.C.S. Istituto Ortopedico Galeazzi, Milan, Italy.
[5] Department of Life Sciences and Biotechnology, University of Ferrara, Ferrara, Italy.
[6] I.R.C.C.S. Ospedale San Raffaele & Università Vita-Salute San Raffaele, Milan, Italy.
[7] Laboratory of Translational Immunology, I.R.C.C.S Istituto Ortopedico Galeazzi, Milan, Italy.

Marco Diani, Fabio Casciano and Laura Marongiu contributed equally to this work.

[*] Corresponding author

Correspondence and requests for materials should be addressed to E.R. (email: eva.reali@grupposandonato.it).

Supplementary Information

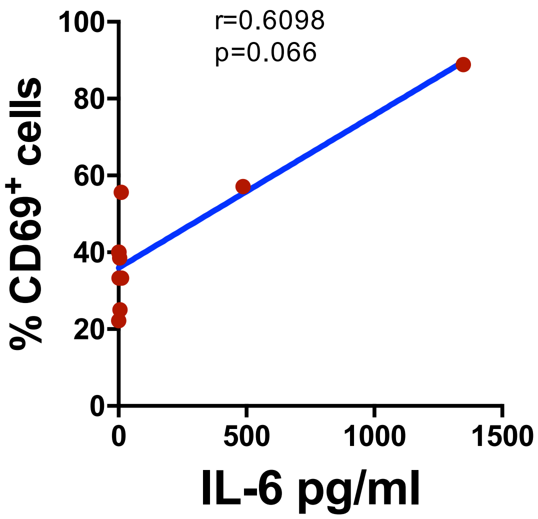

Supplementary Figure 1
Correlation between CD69$^+$ cells in CCR6$^+$CXCR3$^-$CD8$^+$T$_{EMRA}$ cells and IL-6 in patients with psoriatic arthritis.
Sperman correlation analysis between the percentages of CD69$^+$ cells in CCR6$^+$CXCR3$^-$CD8$^+$T$_{EMRA}$ cells and the serum level of IL-6 is reported in the figure.

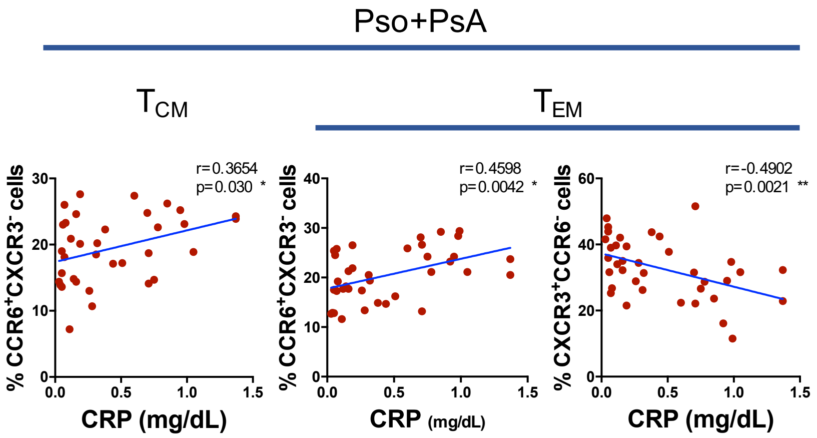

Supplementary Figure 2 Correlation between CXCR3^{-}CCR6^{+} or CXCR3^{+}CCR6^{-} CD4+ circulating T cells and CRP in patients with psoriatic disease.
Correlation between the percentages of CCR6^{+} CXCR3^{-} and CXCR3^{+}CCR6^{-} CD4^{+} T_{EM} or T_{CM} cells and the serum level of C-reactive protein in patients with psoriatic disease (PsO and PsA) is reported in the figure. p values <0.05 were considered significant: * p<0.05.

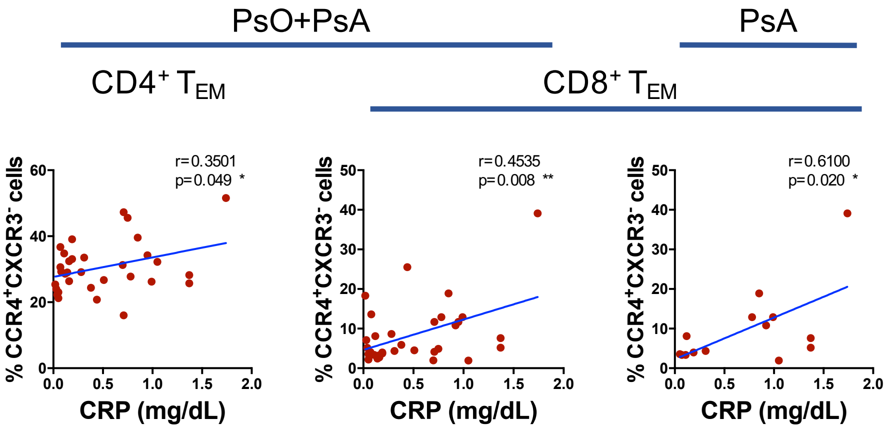

Supplementary Figure 3. Correlation between circulating CCR4$^+$ CXCR3$^-$CD4$^+$ and CD8$^+$ T$_{EM}$ cells and CRP in patients with PsA.

Correlations between the percentages of CCR4$^+$ CXCR3$^-$ CD4$^+$ or CD8 T$_{EM}$ and the serum level of C-reactive protein in patients with either psoriatic disease (PsO and PsA) or with PsA only are reported in the figure. p values <0.05 were considered significant: * $p<0.05$.

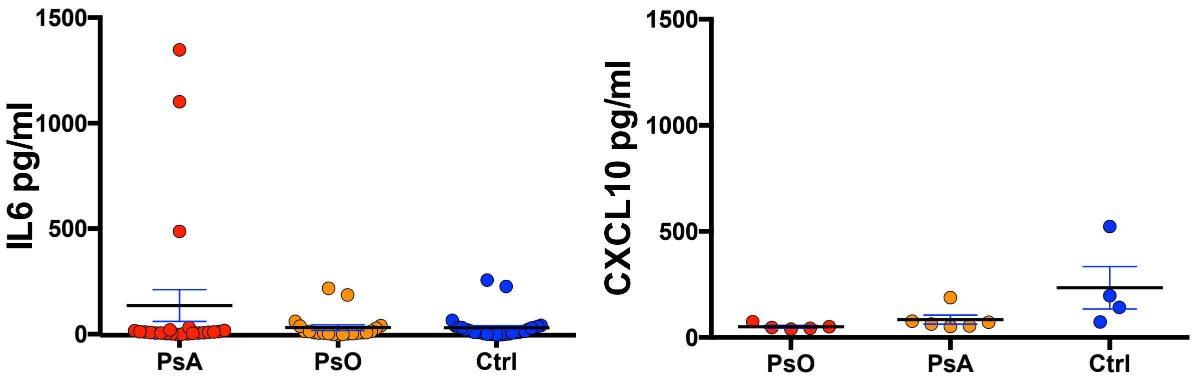

Supplementary Figure 4. Serum level of IL-6 and CXCL10

Serum concentration of CXCL10 and IL6 expressed as pg/ml in PsO and PsA patients and healthy subjects group. Horizontal bar represent mean value ± SEM.

Supplementary materials 1. Panels with antibody and fluorochromes used for the flow cytometric analysis.

Panel 1
CCR4 PE-Cy7
CCR7 PE
CXCR3 PE-Cy5
CD8 APC7
CD4 v500
CD45RA APC
CLA FITC

Panel 2
CCR4 PE-Cy7
CCR7 PE
CXCR3 PE-Cy5
CD8 APC-Cy7
CD4 v500
CD45RA FITC
CD69 AF700

Panel 3
CD4 v500
CD8 APC-Cy7
CD45RA FITC
CCR7 PE
CCR6 PE-Cy7
CCR4 AF647
CCR5 PE-CF594
CD103 BV421

Panel 4
CCR6 PE-Cy7
CCR7 PE
CXCR3 PE-Cy5
CD8 APC-Cy7
CD4 v500
CD45RA APC
CLA FITC

Panel 5
CCR6 PE-Cy7
CCR7 PE
CXCR3 PE-Cy5
CD8 APC-Cy7
CD4 v500
CD45RA FITC
CD69 AF700

Panel 6
CCR7 PE-Cy7
CD45RA FITC
CD4 APC-Cy7
CD8 APC
IFN-γ V450
IL-17A PE

Supplementary Table 1. Characteristics of Patients and Controls

Patient ID	Sex	Age	Disease	Psa duration	PA SI	CRP (mg/dL)	Family history	Comorbidities
PS002	♂	40	Pso	NA	10,5	0,98	Mo	AA
PS003	♂	37	Pso	NA	6,5	0,05	No	None
PS004	♂	24	Pso	NA	7	0,14	Fa	None
PS005	♀	58	Pso	NA	7,5	0,05	No	HC, FP
PS007	♂	56	Pso	NA	12,5	0,71	No	HC, AH, DC
PS008	♀	55	Pso	NA	8,8	0,02	Mo, Gf	None
PS011	♀	43	Pso	NA	8,2	0,51	No	None
PS012	♀	61	Pso	NA	5,1	0,38	Mo	None
PS013	♂	46	Pso	NA	7,5	0,08	No	None
PS015	♀	50	Pso	NA	8,5	0,04	No	None
PS016	♀	63	Pso	NA	12,9	0,07	Mo, Au	RA
PS020	♂	30	Pso	NA	9,5	0,7	No	None
PS023	♂	50	Pso	NA	12	0,71	No	HC
PS024	♀	25	Pso	NA	9	0,03	No	None
PS026	♀	41	Pso	NA	11,4	0,17	Fa	HT
PS031	♂	52	Pso	NA	13,5	0,16	Mo	CH
PS034	♂	20	Pso	NA	19,6	0,19	Mo	None
PS044	♂	60	Pso	NA	19,7	0,95	No	None
PS046	♂	52	Pso	NA	18	0,75	No	D
PS053	♂	61	Pso	NA	10,5	0,44	Gm	HC, D
PS054	♂	53	Pso	NA	20,2	0,16	No	HC
PS063	♂	37	Pso	NA	19,2	0,28	Co	None
PS076	♂	25	Pso	NA	18,4	0,6	Mo	None
PS079	♂	39	Pso	NA	14	0,32	Si, Gm	None
PS080	♂	45	Pso	NA	8	0,06	No	IHD, AH, HC
PS081	♂	59	Pso	NA	15	0,26	No	None
PSA010	♂	47	Pso + PsA	36	1	1,37	No	None
PSA022	♂	45	Pso + PsA	36	3,5	0,34	No	HC
PSA032	♀	45	Pso + PsA	80	10,1	0,85	No	HC
PSA035	♀	63	Pso + PsA	84	14,5	0,78	No	HU
PSA039	♂	48	Pso + PsA	6	0,8	1,74	Br	None
PSA047	♂	42	Pso + PsA	2	12,8	0,05	Mo	None
PSA050	♂	51	Pso + PsA	84	0,8	0,19	No	HC
PSA094	♂	65	Pso + PsA	120	0	0,07	No	HC
PSA059	♂	54	Pso + PsA	180	10	0,12	Mo	AH, DD, HS
PSA060	♂	38	Pso + PsA	12	6,8	1,05	Fa	PP, CH
PSA061	♂	48	Pso + PsA	48	19,3	0,11	No	None
PSA085	♂	54	Pso + PsA	144	9,2	0,31	No	None
PSA088	♂	49	Pso + PsA	2	1,5	0,99	Mo, Da, Ni	AH
PSA090	♀	60	Pso + PsA	3	4	0,92	Fa	AH, HT, RU, FM
PSA091	♂	56	Pso + PsA	120	3	1,37	No	DS, HC

C017	♀	43	None	NA	0	ND	No	None
C018	♂	42	None	NA	0	ND	No	None
C019	♀	38	None	NA	0	ND	No	None
C021	♀	29	None	NA	0	ND	No	None
C025	♂	56	None	NA	0	ND	No	None
C028	♀	51	None	NA	0	ND	No	None
C033	♀	40	None	NA	0	ND	No	None
C036	♀	45	None	NA	0	ND	No	None
C040	♂	55	None	NA	0	ND	No	None
C041	♂	22	None	NA	0	ND	No	None
C042	♂	45	None	NA	0	ND	No	None
C043	♂	41	None	NA	0	ND	No	None
C045	♀	55	None	NA	0	ND	No	None
C048	♀	34	None	NA	0	ND	No	None
C049	♀	34	None	NA	0	ND	No	None
C057	♀	37	None	NA	0	ND	No	None
C058	♀	56	None	NA	0	ND	No	None
CCC	♀	24	None	NA	0	ND	No	None
CLB	♀	23	None	NA	0	ND	No	None
CMD	♂	29	None	NA	0	ND	No	None
C072/ MCCO	♂	57	None	NA	0	ND	No	None
C073/ COSS	♀	37	None	NA	0	ND	No	None
C074/ Tart3	♂	55	None	NA	0	ND	No	None
C077/ LACO	♂	49	None	NA	0	ND	No	None
CAA	♂	38	None	NA	0	ND	No	None
C092	♀	58	None	NA	0	ND	No	None

PsA duration is expressed in months. AH, arterial hypertension; CH, cholelithiasis; D, diabetes; DC, dilated cardiomyopathy; DD, depressive disorder; FM, fibromialgya; FP, familial poliposis; HC, hypercholesterolemia; HS, hepatic steatosis; HT, hypothyroidism; HU, hyperuricemia; IHD, ischemic heart disease; PP, previous pericarditis; RA, rhizarthrosis; RU, recurrent uveitis; Au, aunts; Br, brother; Co, cousin; Da, dauther; Fa, father; Gf, grandfather; Gm, grandmother; Mo, mother; Ni, niece; Si, sister.
